# ﻿A new species of the genus *Lindaspio* Blake & Maciolek, 1992 (Annelida, Spionidae) from a cold seep near Hainan Island, China

**DOI:** 10.3897/zookeys.1153.101406

**Published:** 2023-03-16

**Authors:** Jixing Sui, Dong Dong, Xuwen Wu, Xinzheng Li

**Affiliations:** 1 Laboratory of Marine Organism Taxonomy and Phylogeny, Qingdao Key Laboratory of Marine Biodiversity and Conservation, Institute of Oceanology, Chinese Academy of Sciences, Qingdao 266071, China Institute of Oceanology, Chinese Academy of Sciences Qingdao China; 2 Laboratory for Marine Biology and Biotechnology, Pilot National Laboratory for Marine Science and Technology (Qingdao), Qingdao, China Laboratory for Marine Biology and Biotechnology, Pilot National Laboratory for Marine Science and Technology Qingdao China; 3 University of Chinese Academy of Sciences, Beijing, China University of Chinese Academy of Sciences Beijing China

**Keywords:** Cold seep, *Lindaspiopolybranchiata* sp. nov., Polychaeta, taxonomy

## Abstract

A new species of the spionid genus *Lindaspio* Blake & Maciolek, 1992 was collected from a cold seep near the Hainan Island at a depth of 1758 m. Morphologically, *Lindaspiopolybranchiata***sp. nov.** differs from the congeners in having a narrow, folded caruncle and more neuropodial branchiae (from chaetiger 20). The 18S, COI, and 16S sequences of the new species have been submitted to GenBank. It is the first record of the genus *Lindaspio* from Chinese waters. A key to all species of *Lindaspio* is given.

## ﻿Introduction

Spionidae Grube, 1850 is a group of polychaetes which have a worldwide marine distribution from the intertidal zone to the deep sea, including hydrothermal vent and cold seep environments (Blake and Maciolek 1992; Blake and Ramey-Balci 2020). They are very common and frequently dominant within polychaete communities (Meißner et al. 2014). The genus *Lindaspio* was established by Blake and Maciolek (1992) for *Lindaspiodibranchiata*, which was collected from a hydrothermal mound in the Guaymas Basin (27°01'N, 111°24'W, 2008 m depth). At present, *Lindaspio* comprises three valid species: *L.dibranchiata* Blake & Maciolek, 1992 and *L.southwardorum* Blake & Maciolek, 1992 from a high-heat-flow area in the Middle Valley segment of the Juan de Fuca Ridge (48°25.8'N, 128°40.9'W, 2425 m depth), as well as *L.sebastiena* Bellan, Dauvin & Laubier, 2003 from an oil field off Congo (5°16.390'N, 11°33.848'W, 150 m depth).

**Figure 1.**
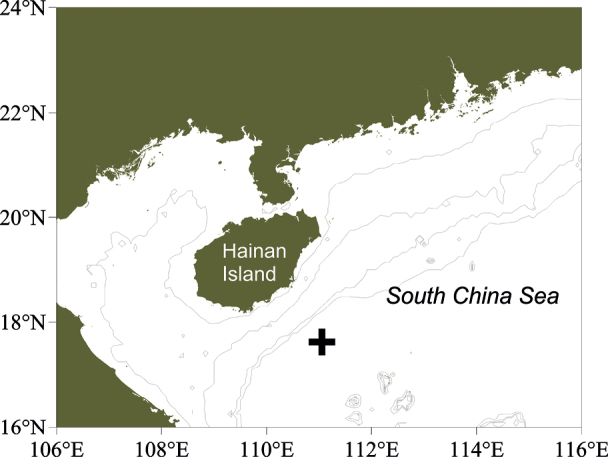
Sampling site of *Lindaspiopolybranchiata* sp. nov.

In May 2021, a biodiversity survey in the area of a cold-seep (named “Lingshui”) near Hainan Island was conducted by the Institute of Oceanology, Chinese Academy of Sciences (IOCAS) using the R/V Kexue. During the cruise, some specimens of an undescribed species of polychaetes were collected by the ROV Faxian at a depth of 1758 m (Fig. 1).

## ﻿Materials and methods

All the specimens were fixed and preserved in 75% ethanol solution and are deposited in the Marine Biological Museum of the Chinese Academy of Sciences (**MBMCAS**) in the Institute of Oceanology, Chinese Academy of Sciences (**IOCAS**). The specimens were observed, measured, and photographed with a Nikon AZ100 stereomicroscope. The fine morphology of anterior end, notochaetae, and neurochaetae was observed using a Hitachi S-3400N scanning electron microscope (SEM).

Total DNA was extracted with the DNeasy Blood and Tissue Kit (Qiagen, Hilden, Germany) and stored at −20 °C. Partial fragments of COI (~700 bp), 16S (~450 bp), and 18S (~1800 bp) genes were amplified by the polymerase chain reaction (PCR) using primers polyLCO/polyHCO (Carr et al. 2011), 16S-AnnF/AnnR (Sjölin et al. 2005), and 18S-A/B (Medlin et al. 1988), respectively. Amplifications were carried out in a reaction mixture containing 2 μl of template DNA, 12.5 μl of Premix TaqTM (Takara, Otsu, Shiga, Japan), 0.5 μl of each primer (10 mM), and sterile distilled H_2_O to a total volume of 25 μl with cycling conditions as follows: initial denaturation at 94 °C for 10 min, followed by 35–45 cycles of denaturation at 94 °C for 30 sec, annealing at 45 °C for 40 sec, and extension at 72 °C for 90 sec. A final extension at 72 °C for 5 min was included. PCR products with distinct bands after electrophoresis on 1.5% agarose gels were sent to Qingke Laboratory (Qingdao, China) for sequencing using the same set of primers that were used for PCR amplifications. Fragments with overlapping sequences (forward and reverse) were merged into consensus sequences using CONTIG EXPRESS (a component of Vector NTI Suite 6.0, Life Technologies, Carlsbad, CA, USA). The assembled sequences were checked by BLASTing in GenBank to ensure that the DNA was not contaminated. Finally, all the new sequences were submitted to GenBank.

## ﻿Systematics

### ﻿Taxonomy

**Family Spionidae Grube, 1850**

#### ﻿Genus Lindaspio

Taxon classificationAnimaliaSpionidaSpionidae

Blake & Maciolek, 1992

C80C04D1-E702-5622-A1DE-5E6AC102E09F

##### Type species.

*Lindaspiodibranchiata* Blake & Maciolek, 1992.

##### Generic diagnosis

**(according to Gérard et al. 2003).** Prostomium incised, developed into two frontal lobes or weak horns; caruncle present or absent; occipital tentacle absent. Peristomial wings absent. Notopodia of chaetigers 2–4 with fascicles of heavy spines. Anterior neuropodial spines present. Dorsal branchiae starting from chaetiger 2; ventral branchiae starting from an anterior segment, branchiae closely associated with parapodial lamellae, continuing to posterior end. Chaetiger 1 reduced, with notopodia reduced to single lamellae lacking notochaetae. Following notopodia and neuropodia with capillaries and hooded hooks. Pygidium simple, conical, lacking cirri.

#### ﻿ Lindaspio polybranchiata sp. nov.

Taxon classificationAnimaliaSpionidaSpionidae

DCA70EAA-E387-5B90-B38D-1CA415CE0CD4

https://zoobank.org/601C3193-EE40-4088-9029-2CB1BDDC8641

Figs 2 , 3 , 4

##### Material examined.

***Holotype***: Complete, Lingshui cold seep cruise, Faxian Dive 252, 1758 m, 17°37'N, 111°03'E, coll. crew of R/V Kexue, 28 May 2021, MBM 304666. ***Paratypes***: 4 specimens, complete, same collection data as holotype, MBM 304662-MBM 304665.

***Non-type***: 14 incomplete specimens depositing in one specimen bottle, MBM304661.

##### Description of holotype.

Total length 55 mm, maximum width 4 mm, including chaetae. More than 350 crowded chaetigers. Color pale in alcohol.

**Figure 2.**
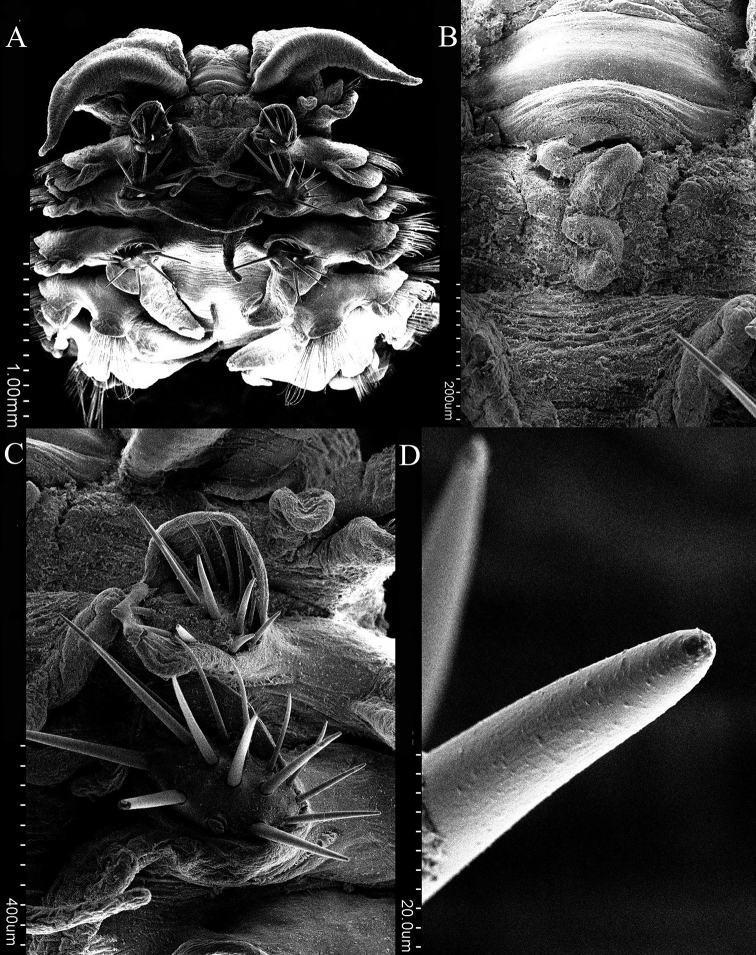
*Lindaspiopolybranchiata* sp. nov. (Paratype, MBM 304662) **A** anterior end, dorsal view **B** caruncle **C** modified notopodial spines from anterior chaetiger **D** tip of modified notopodial spine.

**Figure 3.**
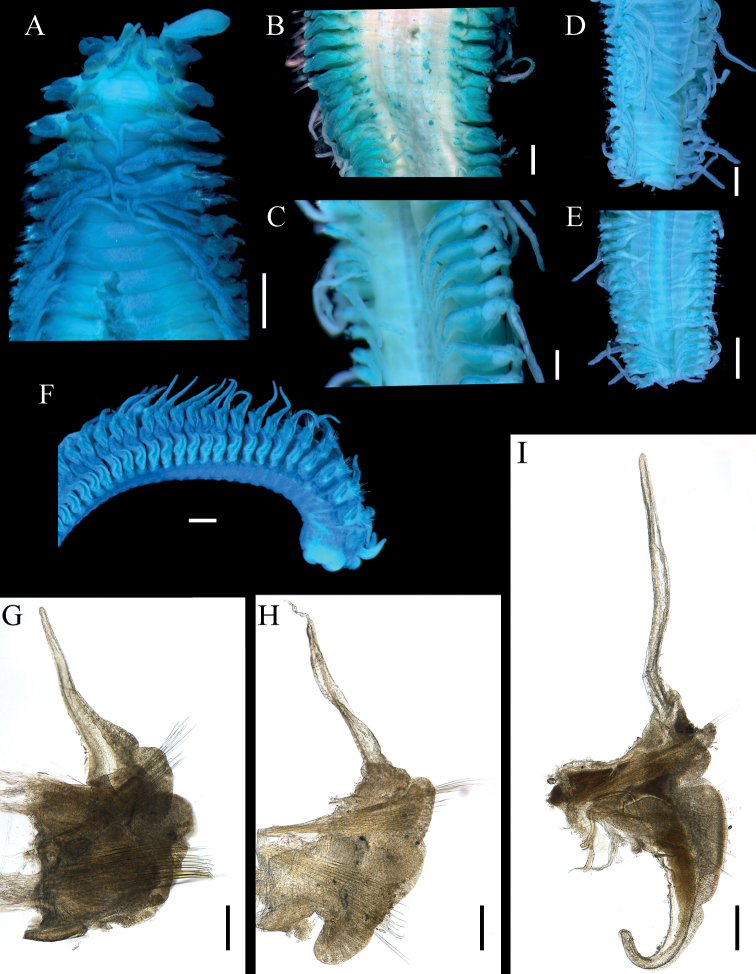
*Lindaspiopolybranchiata* sp. nov. (Paratype, MBM 304663) **A** anterior end in dorsal view **B** anterior segments in ventral view, starting from segment 20 **C** median segments in ventral view **D** posterior end in dorsal view **E** posterior end in ventral view **F** anterior end in lateral view **G** parapodium from segment 12 **H** parapodium from segment 31; I. parapodium from segment 51. Scale bars: 1 mm (**A, E**); 500 µm (**B–D, F**); 250 µm (**G–I**).

Prostomium anteriorly bilobed, forming two broadly swollen lobes (Fig. 2A), continuing posteriorly as narrow, undulating caruncle to anterior margin of chaetiger 2 (Fig. 2B). Palps short, thick, tapering to pointed tip, not extending beyond chaetiger 3 (Figs 2A, 3A). Chaetiger 1 reduced, notopodia reduced to flattened lamella, lacking notochaetae (Fig. 3A); neuropodia with well-developed pre- and postchaetal lamellae (Fig. 3F) and fascicles of capillaries. Notopodia of chaetigers 2–4 shifted dorsally to medial position, with pre- and postchaetal lamellae forming three rows enclosing cluster of modified spines (Figs 2C, D, 3A, F). Notopodia of chaetigers 5–8 gradually shifted to lateral position (Fig. 3A, F). Notopodia from chaetiger 5 and neuropodia from chaetiger 2 with well-developed pre- and postchaetal lobes.

**Figure 4.**
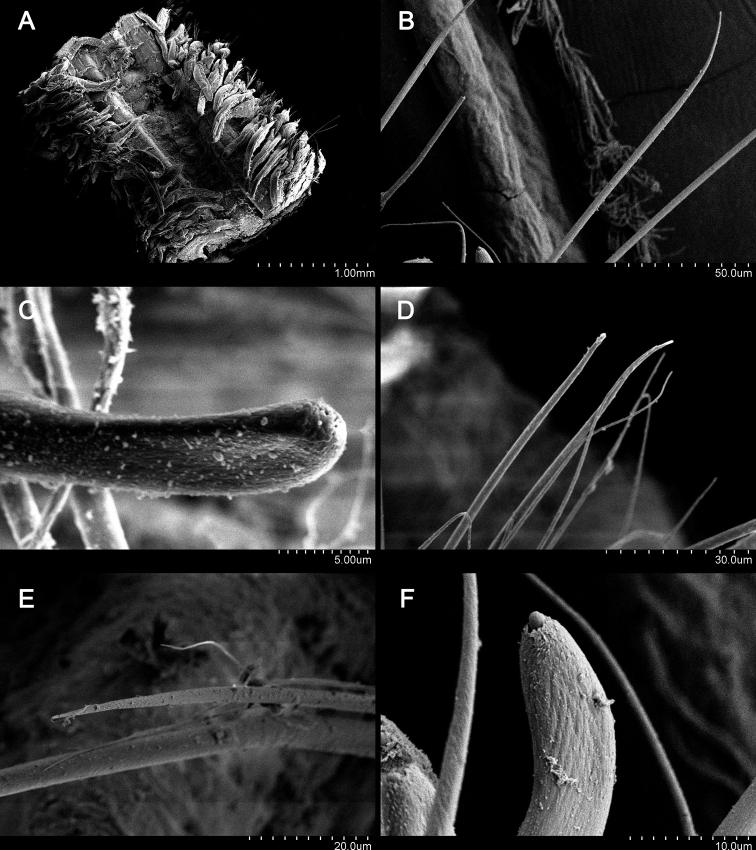
*Lindaspiopolybranchiata* sp. nov. (Paratype, MBM 304662) **A** ventral view, middle part **B** anterior notochaetae **C** notopodial hooded hooks **D** anterior neurochaetae **E** details of neurochaetae **F** neuropodial hooded hook.

Dorsal branchiae fingerlike, appear from chaetiger 2 (Fig. 3A) and remaining short to chaetiger 30, thereafter branchiae becoming thinner, longer, extending to dorsal midline. Ventral branchiae absent in the anterior part (Fig. 3G). From chaetiger 20 small neuropodial expansion (Fig. 3B), becoming well-developed neuropodial branchiae (Fig. 3C) at chaetiger 40, until about chaetiger 80, becoming longer, more cylindrical, nearly reaching ventral midline, but never as long as dorsal branchiae (Figs 3H, I, 4A).

Notochaetae of chaetigers 2–4 modified into cluster of about 15 heavy spines (Figs 2C, 3A); subsequent notochaetae numerous, thin capillaries (Fig. 4B) until about chaetiger 60 where hooded hooks appear; capillaries become heavier and more limbate in far posterior segments; individual notopodial hooks strongly curved, with pointed tips without minute teeth (Fig. 4C).

Anterior neurochaetae include row of heavy spines and ventral bundle of thin capillaries (Fig. 4D); neuropodial spines each with smooth shaft that tapers gradually, then continues as fine, pointed tip with fine bristles sometimes visible along edge (Fig. 4E); spines present until about chaetiger 40, then accompanied with thin capillaries; these capillaries accompanied by neuropodial hooded hooks from about chaetiger 65; each hook smaller, more delicate than notopodial hooks, without minute teeth (Fig. 4F). Pygidium simple, conical, lacking cirri (Fig. 3D, E).

GenBank Accession Number: COI OQ582086, 16S OQ592054, 18S OQ592055.

##### Remarks.

As mentioned above, three species have been reported in the genus *Lindaspio*. The new species is easily distinguished from *L.dibranchiata* and *L.sebastiena* in having a narrow, undulating caruncle, while *L.sebastiena* has no caruncle and *L.dibranchiata* has a short, mounded caruncle. The new species resembles *L.southwardorum* in having similar caruncle, ventral branchiae and dorsal clusters of spines in anterior notopodia, while the latter differs in having first neuro- branchiae from chaetiger 55 (vs. chaetiger 20 in the new species) and having more modified spines on chaetigers 2–4 (20 vs. 15).

The BLAST percent identity of the 18S sequence between the new species and *Lindaspiodibranchiata* is 99% (1758/1762 bp), suggesting that they are congeneric. Additionally, the COI and 16S sequences of our specimen are identical to those of *Lindaspio* sp. (GenBank accession number OK032597.1), which confirms that the unverified species of *Lindaspio* in GenBank is a *Lindaspiopolybranchiata* sp. nov.

##### Etymology.

The species is so named because it has more neuropodial branchiae than the congeners.

##### Distribution.

Currently only known from the type locality, near Hainan Island, China, at a depth of 1758 m (Fig. 1).

### ﻿Key to *Lindaspio* species

**Table:** 

1	Caruncle absent	***L.sebastiena* Bellan, Dauvin & Laubier, 2003**
–	Caruncle present	**2**
2	Caruncle oval	***L.dibranchiata* Blake & Maciolek, 1992**
–	Caruncle undulating	**3**
3	First neuro- branchiae starting from about chaetiger 55	***L.southwardorum* Blake & Maciolek, 1992**
–	First neuro- branchiae starting from about chaetiger 20	***L.polybranchiata* sp. nov.**

## Supplementary Material

XML Treatment for Lindaspio

XML Treatment for Lindaspio polybranchiata

## References

[B1] Blake JA Maciolek NJ (1992) Polychaeta from deep-sea hydrothermal vents in the Eastern Pacific. III: A new genus and two new species of Spionidae from the Guaymas Basin and Juan de Fuca Ridge with comments on a related species from the western North Atlantic. Proceedings of the Biological Society of Washington 105(4): 723–732.

[B2] Blake JA Ramey-Balci PA (2020) A new genus and species of spionid polychaete (Annelida, Spionidae) from a deep-water cold seep site in the eastern Mediterranean sea off Turkey. Zoosymposia 19(1): 121–134. 10.11646/zoosymposia.19.1.14

[B3] Carr CM Hardy SM Brown TM Macdonald TA Hebert PD (2011) A tri-oceanic perspective: DNA barcoding reveals geographic structure and cryptic diversity in Canadian polychaetes. PLoS ONE 6(7): e22232. 10.1371/journal.pone.0022232 PMC3136506 21829451

[B4] Gérard B Jean-Claude D Lucien L (2003) The genus *Lindaspio* (Annelida: Polychaeta: Spionidae), and a new species from an oil field off Congo, western Africa. Journal of Natural History 37(20): 2413–2424. 10.1080/00222930210155666

[B5] Medlin L Elwood HJ Stickel S Sogin ML (1988) The characterization of enzymatically amplified eukaryotic 16S-like rRNA-coding regions. Gene 71(2): 491–499. 10.1016/0378-1119(88)90066-2 3224833

[B6] Meißner KBA Guggolz T Götting G (2014) Spionidae (Polychaeta: Canalipalpata: Spionida) from seamounts in the NE Atlantic. Zootaxa 3786(3): 201–245. 10.11646/zootaxa.3786.3.1 24869536

[B7] Sjölin E Erséus C Källersjö M (2005) Phylogeny of Tubificidae (Annelida, Clitellata) based on mitochondrial and nuclear sequence data. Molecular Phylogenetics and Evolution 35(2): 431–441. 10.1016/j.ympev.2004.12.018 15804413

